# Combined monophosphoryl lipid A and alpha-Galactosyl ceramide adjuvants enhance the systemic immune response to intranasal inactivated influenza vaccination in a murine homologous challenge model

**DOI:** 10.1371/journal.pone.0362356

**Published:** 2026-11-11

**Authors:** Thi Len Ho, Thanh Tam Pham, Eun-Ju Ko

**Affiliations:** 1 Interdisciplinary Graduate Program in Advanced Convergence Technology and Science, Jeju National University, Jeju, Republic of Korea; 2 Department of Veterinary Medicine, College of Veterinary Medicine, Jeju National University, Jeju, Republic of Korea; 3 Veterinary Medical Research Institute, Jeju National University, Jeju, Republic of Korea; Indian Institute of Chemical Technology, INDIA

## Abstract

Inactivated influenza vaccines (IIVs) often fail to provide robust mucosal protection, necessitating the development of potent adjuvants for intranasal delivery. This study evaluated a novel adjuvant combination of Monophosphoryl lipid A (MPL), a TLR4 agonist, and alpha-Galactosyl Ceramide (α-GalCer), an NKT cell ligand, to enhance the efficacy of an intranasal inactivated A/PR8 (iPR8) influenza vaccine. *In vitro* results demonstrated that MPL+ α-GalCer increased the frequencies of CD4⁺ CD25⁺ , CD8⁺ CD25⁺ and CD4⁺ CD69⁺ T cells relative to both the vehicle control and either single adjuvant in splenocyte cultures, whereas in purified dendritic cell and macrophage cultures MPL+ α-GalCer was no more effective than MPL alone. Splenocyte supernatants showed higher elevated TNF- α, IL-2, IFN-γ, IL-4, and IL-13. This pre-activation drove robust allogeneic T-cell proliferation in a mixed lymphocyte reaction, indicating enhanced allostimulatory capacity rather than influenza-specific priming. *In vivo*, a single intranasal dose of iPR8+ MPL+ α-GalCer elicited high systemic IgG titers and was effective at inducing Th1-associated IgG2a alongside IgG1. Following an LD50-range PR8 challenge, the combined-adjuvant group showed 100% survival, which was not statistically significant and lower lung viral titers at 7 days post-infection. This group exhibited elevated post-challenge lung-associated antigen-specific IgA and a reduced viral burden, which was accompanied by lower inflammatory cell infiltration and cytokine levels. The protection of combination group was accompanied by increased frequencies of ex vivo antigen-responsive CD4^+^ and CD8^+^ T cells recovered post-challenge. Our findings suggest that the MPL+ α-GalCer combination enhances innate and adaptive immune responses, providing an effective adjuvant platform for homologous mucosal protection in a murine model.

## Introduction

Seasonal influenza A virus (IAV) remains a persistent global public health threat, causing significant morbidity, mortality, and economic disruption annually [1]. While vaccination is the most effective strategy for preventing influenza, the efficacy of current vaccines, particularly standard inactivated influenza vaccines (IIVs), can be suboptimal [2]. These vaccines are often poorly immunogenic in high-risk populations and typically induce strain-specific systemic antibodies (IgG) with limited capacity to establish robust, cross-protective immunity at the respiratory mucosa, the primary site of infection.

Intranasal vaccination is an attractive alternative because it mimics the natural route of infection, which can build strong immunity throughout the body and at the site of infection [3–7]. However, inactivated viral antigens, such as the A/Puerto Rico/8/34 (PR8) virus used in this study, are often poorly immunogenic when delivered mucosally and require potent adjuvants to elicit a protective immune response [8–10]. Therefore, the development of novel adjuvant combinations that can safely and effectively enhance the immunogenicity of intranasal vaccines is a critical research priority.

This study focuses on combining two distinct classes of adjuvants: Monophosphoryl lipid A (MPL) and alpha-Galactosyl Ceramide (α-GalCer). MPL, a detoxified derivative of lipopolysaccharide, is a well-established TLR4 agonist known to be a potent activator of antigen-presenting cells (APCs) like dendritic cells (DCs) and macrophages, primarily driving Th1-biased immune responses [11,12]. In contrast, α-GalCer is a glycolipid ligand that specifically activates invariant Natural Killer T (NKT) cells via the CD1d molecule [13,14]. Activated NKT cells serve as a critical bridge between innate and adaptive immunity, rapidly producing cytokines and providing co-stimulation to B cells and T cells [15,16].

While the individual properties of MPL and α-GalCer are well-documented, a clear rationale supports evaluating whether their combination yields an enhanced combined response through complementary innate and adaptive immune pathways. MPL predominantly activates APCs through the TLR4-TRIF and MyD88 pathways, driving Th1-skewed cytokine production (IL-12p70, TNF-α, IFN-β) [17]. Complementarily, α-GalCer is a well-known glycolipid ligand reported in the literature to stimulate invariant NKT (iNKT) cells via CD1d presentation [18]. We hypothesize that combining MPL and α-GalCer promotes complementary innate and adaptive immune activation, where TLR4-driven APC maturation and glycolipid-mediated T/NKT-like cell activation cooperatively enhance overall immunogenicity. Prior mechanistic literature strongly supports this model: De Becker et al. demonstrated that MPL increases the antigen-presenting function of APCs [19], while Fujii et al. showed that α-GalCer-stimulated iNKT cells rapidly induce the full maturation of DCs *in vivo* [20]. Consequently, the combination of these ligands allows the elevated IL-12 produced by MPL-stimulated APCs to reciprocally enhance and amplify NKT-mediated IFN-γ production, thereby creating a cooperative, highly potent, and eliciting both cellular and humoral responses.

In this research, we evaluated the adjuvant effects of MPL, α-GalCer, or their combination (MPL+ α-GalCer) on an intranasal inactivated PR8 (iPR8) vaccine. *In vitro*, we assessed APCs and splenocyte activation alongside allogeneic T-cell proliferation. *In vivo*, we utilized a single-dose mouse model to determine systemic antibody responses and protective efficacy against a LD50-range A/PR8 challenge, concluding with lung and BAL fluid analysis to quantify pulmonary inflammatory indices.

## Materials and methods

### Animals and ethics

Female BALB/c and C57BL/6 mice, aged 6–8 weeks, were purchased from SamTako (Korea). All animal experimental protocols in this study were reviewed and approved by the Institutional Animal Care and Use Committee (IACUC) of Jeju National University, Jeju, Korea (Protocol Number: 2025−0076). All methods were carried out in accordance with relevant guidelines including the ARRIVE 2.0 guidelines [21].

Mice were housed under specific-pathogen-free conditions in individually ventilated cages (4 mice per cage) at 22 ± 2 °C and 50 ± 10% relative humidity on a 12 h light/dark cycle, with irradiated pelleted food and autoclaved water available *ad libitum* and were acclimatized for 14 days before any procedure.

Anaesthesia: All intranasal and retro-orbital procedures were performed under isoflurane anaesthesia (2–3% in oxygen via calibrated vaporizer), with depth confirmed by loss of pedal withdrawal reflex. Animals recovered on a warming pad.

Analgesia: Systemic analgesics were withheld per IACUC protocol. Suffering was minimized by daily weight and clinical scoring, provision of nesting material, and placement of softened chow and hydration gel on the cage floor from onset of weight loss. Cages were inspected twice daily during peak morbidity (days 5–9).

Humane endpoints and euthanasia: Predefined endpoints were ≥ 20% body-weight loss or severe clinical distress. Animals meeting either criterion were euthanized immediately and recorded as non-survivors. All euthanasia, humane endpoint or scheduled (day 7), was by gradual-fill CO_2_ (30–70% chamber volume/min, ≥ 1 min post-apnoea) followed by cervical dislocation, per AVMA Guidelines (2020) [22] and IACUC policy.

### Reagents

Monophosphoryl Lipid A (MPL; synthetic TLR4 agonist) was purchased from InvivoGen (Cat. # vac-mpla, USA). α-Galactosylceramide (α-GalCer) was obtained from Sigma-Aldrich (Cat. # 867000P, USA). Madin-Darby Canine Kidney (MDCK) cells (Cat. # 10034, Korean Cell Line Bank, South Korea) were cultured in Dulbecco’s Modified Eagle Medium (DMEM; Gibco, Thermo Fisher Scientific) supplemented with 10% fetal bovine serum (FBS; Corning) and 1 × Antibiotic-Antimycotic (Gibco). Influenza A/Puerto Rico/8/34 (H1N1; PR8) virus strain (Cat. # VR-1469, ATCC, USA), the virus stock was at passage 3; a single batch of iPR8 was used for all experiments; antigen content was standardized by BCA total protein was used for all experimental challenges and antigen preparations.

MPL stock solutions (1 mg/mL) were prepared by dissolving in DMSO. α-GalCer stock solutions 1 mg/mL were dissolved in DMSO, heated at 60 °C for 10 minutes to completely clarified. Stock solutions were stored in single-use aliquots at −20 °C. For *in vitro* experiments, stocks were diluted in complete culture medium to the working concentrations indicated, giving a final DMSO concentration of 0.005% (v/v); vehicle control wells received medium containing the same final DMSO concentration (0.005%, v/v). On the day of immunization, adjuvants were diluted into sterile phosphate-buffered saline (PBS) containing inactivated PR8 antigen and the final DMSO concentration in the 50 µL intranasal dose was 2% (v/v).

The iPR8 vaccine was prepared by propagating the A/Puerto Rico/8/34 (PR8) virus in Madin-Darby canine kidney (MDCK) cells as described previously [23]. Briefly, MDCK cells were maintained in DMEM (Gibco) supplemented with 10% FBS (Corning) and 1 × Antibiotic-Antimycotic (Gibco). Monolayers were washed with PBS and infected with PR8 at an MOI of 0.01 in serum-free DMEM containing TPCK-treated trypsin (1 µg/mL; Sigma-Aldrich). After 1 h adsorption at 37 °C in 5% CO_2_, the inoculum was replaced with fresh infection medium, and cultures were incubated for 3-4 days until cytopathic effect (CPE) was evident. Supernatants were clarified (1,500 rpm, 20 min), inactivated with 1% formalin at 4 °C, overnight, concentrated by ultracentrifugation (30,000 rpm, 1 h), and resuspended in PBS. Protein concentration was determined by BCA assay (DoGenBio), and iPR8 stocks were stored at −80 °C until use. Complete viral inactivation was verified as shown in S1 Fig.

### Immunization and viral challenge

Female BALB/c mice were allocated into two distinct experimental cohorts to avoid confounding survival endpoints with terminal sampling: (1) Morbidity and Mortality Cohort (Cohort 1, n = 4 mice/group): Immunized intranasally (i.n.) under oxygen-controlled isoflurane anesthesia with a single 50 µL dose of iPR8 (1 µg) alone or adjuvanted with MPL (1 µg), α-GalCer (1 µg), or MPL (1 µg) + α-GalCer (1 µg) in PBS. Two weeks post-vaccination, mice in Cohort 1 (*n* = 4/group) were challenged intranasally with 100 PFU of influenza A/PR/8/34 virus in 50 μL PBS under oxygen-controlled isoflurane anesthesia. A dose of 100 PFU represents an approximate LD50 challenge in female BALB/c mice, as previously established to induce severe pulmonary disease, significant weight loss, and ∼50% mortality [24,25]. Animals were monitored daily for 14 days for body weight changes and survival. Humane endpoints were set as body weight loss exceeding 20%, at which point animals were humanely euthanized by CO_2_ asphyxiation followed by cervical dislocation and recorded as non-survivors on that day; (2) Terminal Sampling Cohort (Cohort 2, n = 4 mice/group): Immunized and challenged identically to Cohort 1. Serum was collected 2 weeks post-vaccination (pre-challenge) via retro-orbital blood sampling for antibody titration. At 7 dpi, all mice in Cohort 2 were sacrificed for collection of blood, bronchoalveolar lavage (BAL) fluid, lung tissue, and spleen.

### Flow cytometry

All cell samples, including both *in vitro* cultures and *in vivo*-derived Bal, lungs, spleen cells, bone-marrow were first incubated with TruStain FcX™ (anti-mouse CD16/32) antibody (clone 93, BioLegend) to prevent non-specific antibody binding. For surface staining, cells were then incubated with antibodies (listed in S1 Table) to assess the activation of DCs, macrophages, T cells, and inflammatory cells following the gating strategies in S7–S10 Figs. For intracellular cytokine staining, cell were stimulated with inactivated A/PR/8/34 virus (iPR8, 2 µL/mL) in the presence of BD GolgiStop™ solution (1 x working concentration; BD Biosciences) for 4 h at 37 °C in 5% CO_2_. Parallel wells cultured with medium alone served as unstimulated controls, and wells treated with Cell Stimulation Cocktail (Invitrogen™) served as positive controls. Identical stimulation conditions were used for lung and splenic cells. Following stimulation, these cells were Fc-blocked, stained for surface markers, and then fixed and permeabilized using the Fixation/Permeabilization Solution Kit (BD Biosciences) prior to intracellular cytokine staining.

Data were acquired using a Beckman CytoFLEX LX at the Bio-Health Materials Core Facility, Jeju National University, and analyzed using FlowJo Software (Tree Star Inc.).

All flow cytometry data are reported as cell frequencies within indicated parent gates. Because total tissue cell yields were not recorded, absolute cell numbers were not derived. Changes in cell frequencies reflect shifts in population proportions rather than absolute cell expansion.

### ELISA

Immune sera were collected two weeks post-vaccination to measure antigen-specific antibody levels. Briefly, 96-well ELISA plates were coated with inactivated A/PR8 (iPR8, 400 ng/well) and incubated overnight at 4°C. The plates were then blocked using 3% bovine serum albumin (BSA) in PBST (PBS containing 0.05% Tween 20). Sera were diluted 1:100 and added to the plates and incubated for 2 hours. After washing with PBST, bound antibodies were detected using horseradish peroxidase (HRP)-labeled secondary antibodies specific for mouse IgG, IgG1, and IgG2a. The reaction was developed using a tetramethylbenzidine (TMB) substrate, and absorbance was measured at 450 nm using a BioTek Synergy LX Multi-Mode Reader (USA).

Cytokine and chemokine levels in cell culture supernatants, lung tissue homogenates, and bronchoalveolar lavage (BAL) fluid were measured using commercial ELISA kits as detailed in S2 Table. Cell culture supernatants were diluted 1:5 in assay diluent prior to analysis. For tissue samples, individual lungs were homogenized in 2 mL of RPMI-1640 medium supplemented with antibiotics, and BAL fluid was harvested by flushing the lungs with 1.4 mL of PBS. Lung homogenates and BAL fluid supernatants were used undiluted directly for ELISA assays according to the manufacturers’ instructions. Because each sample represents one complete lung processed in an identical volume, the reported concentrations correspond to the total cytokine content of a whole lung and are not influenced by variation in the amount of tissue sampled. Lung wet weights were not recorded prior to homogenization, and total protein concentrations in the homogenates were not determined; cytokine and chemokine values are therefore reported as absolute concentrations (pg/mL) without normalization to tissue weight or total protein, which limits direct quantitative comparison with other studies.

### Virus titration

Viral titers were determined by plaque assay on confluent MDCK cell monolayers in 6-well plates. Cells were washed with sterile PBS and inoculated with 1mL of 10-fold serial virus dilutions prepared in serum-free DMEM. Following a 1 h adsorption at 37°C and 5% CO_2_, the inoculum was aspirated and replaced with an overlay medium consisting of a 1:1 mixture of 2x DMEM and 2% low-melt agarose, supplemented to a final concentration of 1 µg/mL TPCK-treated trypsin. Plates were incubated for 3 days at 37°C, 5% CO_2_ to allow plaque formation. Finally, the cells were fixed with 4% formalin, the overlay was removed, and the monolayers were stained with 0.1% crystal violet to visualize and count plaques. Titers were calculated based on the plaque count, dilution factor, and inoculum volume, and were expressed as plaque-forming units (PFU)/mL. Moreover, lung homogenates were titrated in 10-fold serial dilutions, with 1.0 mL of the lowest dilution (10^−2^) inoculated per well in duplicate. The limit of detection was therefore 1 plaque per well, corresponding to 100 PFU/mL of homogenate (200 PFU per lung in 2 mL total homogenate; log_10_ = 2.00). Samples with no plaques at the lowest dilution were assigned a value equal to the limit of detection (100 PFU/mL; log_10_ = 2.00) for statistical analysis.

### Hemagglutination inhibition (HAI) assay

Immune sera collected 2 weeks post-vaccination were treated with receptor-destroying enzyme (RDE; Sigma-Aldrich) for 18 h at 37 °C, then heat-inactivated at 56 °C for 30 min. RDE-treated sera were serially diluted in PBS, incubated with 4 HA units of PR8 virus for 30 min at room temperature, and mixed with 0.5% chicken red blood cells (RBCs). After settling, hemagglutination was scored. The HAI titer was the highest serum dilution that completely inhibited hemagglutination. Positive (homologous anti-PR8 serum) and negative (naive mouse serum) controls, together with a virus back-titration and an RBC-only control, were included on every plate. The assay limit of detection was a titer of 10; sera showing no detectable inhibition at the starting dilution were assigned a titer of 5 (half the limit of detection). Titers were log2-transformed for statistical analysis and are reported as geometric mean titers with individual animal values shown (Fig 3B).

**Fig 1:**
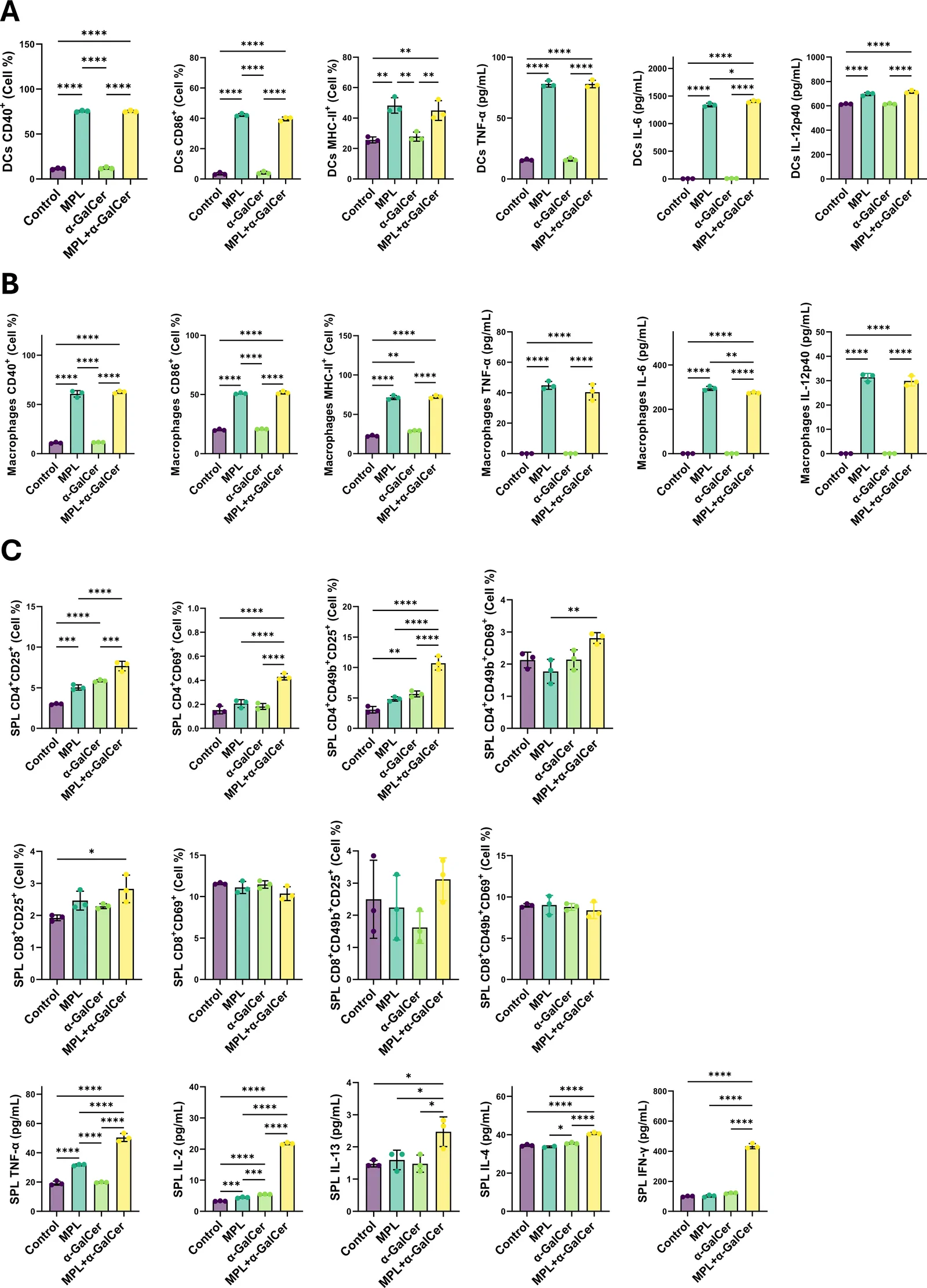
*In vitro* activation and cytokine secretion by immune cells following adjuvant treatment. Bone marrow-derived dendritic cells (DCs) (A), macrophages (B), and splenocytes (C) were cultured for 48 hours with MPL (0.05 µg/mL), α-GalCer (0.05 µg/mL), or a combination of MPL and α-GalCer. After treatment, cells were analyzed by flow cytometry to assess the expression of activation markers, and culture supernatants were collected to quantify cytokine production levels by ELISA. All data are expressed as the mean ± SD. Statistical analysis was performed using a one-way ANOVA followed by Tukey’s multiple comparisons test (*p < 0.05, **p < 0.01, ***p < 0.001, ****p < 0.0001 relative to the indicated group). All experiments were performed independently three times (N = 3 independent biological experiments) with comparable results.

**Fig 2:**
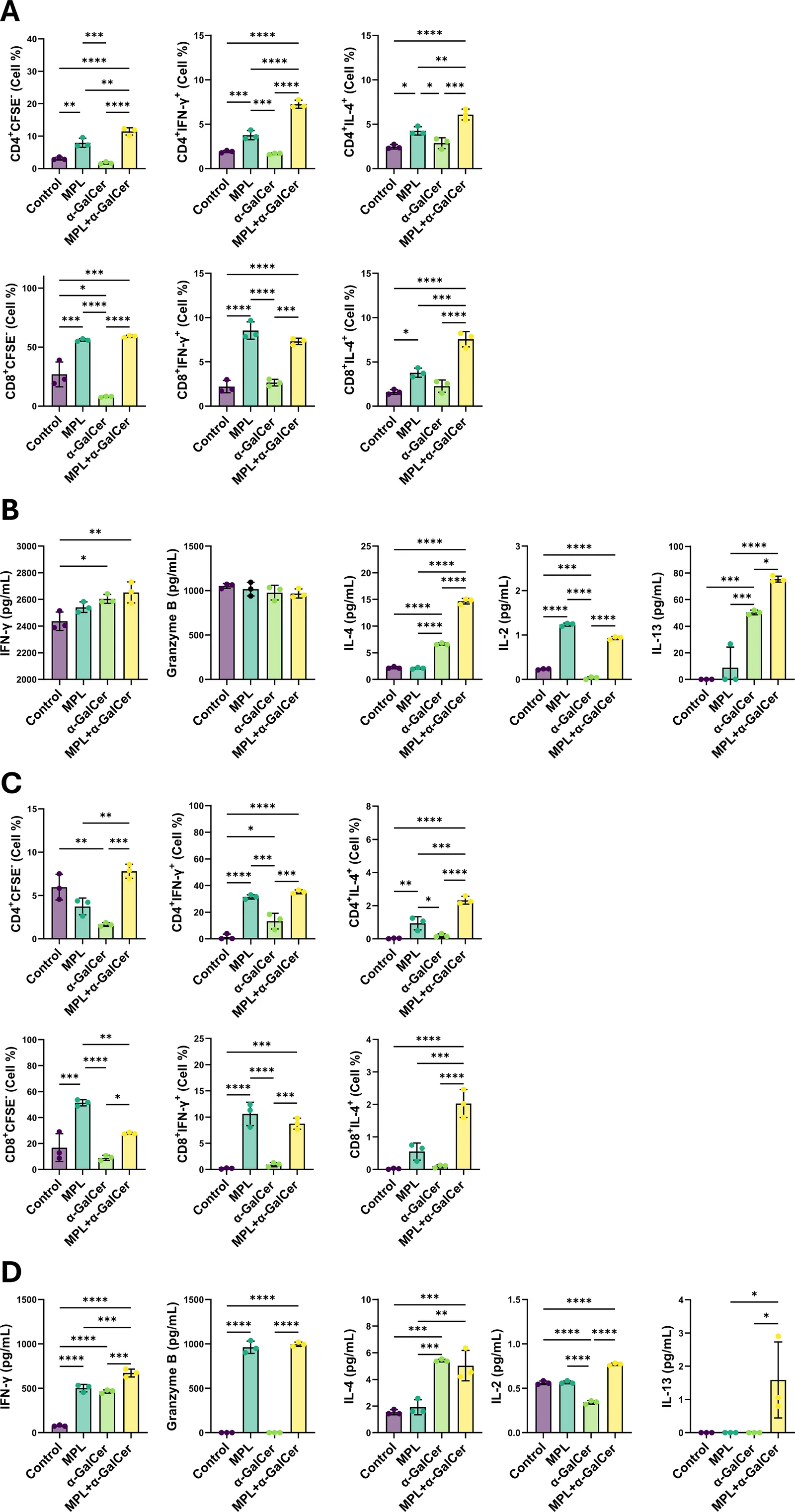
Proliferation of T cells co-cultured with adjuvant-pre-treated DCs and macrophages. Bone marrow-derived dendritic cells (DCs) and macrophages were pre-treated for 2 days with MPL (0.05 µg/mL), α-GalCer (0.05 µg/mL), or their combination. Allogeneic lymphocytes were harvested from spleen and labeled with CFSE. These CFSE-labeled lymphocytes were then co-cultured for 5 days with the pre-activated DCs (A, B) or macrophages (C, D). T cell proliferation was determined by flow cytometry, and cytokines were measured by ELISA and flow cytometry. All data are expressed as the mean ± SD. Statistical analysis was performed using a one-way ANOVA followed by Tukey’s multiple comparisons test (*p < 0.05, **p < 0.01, ***p < 0.001, ****p < 0.0001 relative to the indicated group). All experiments were performed independently three times (N = 3 independent biological experiments) with comparable results.

### *In vitro* stimulation of APCs and splenocytes

Primary bone marrow-derived dendritic cells (DCs), bone marrow-derived macrophages, and splenocytes were prepared as described previously [26] from naïve BALB/c donor mice (n = 3 mice pooled per cell preparation per experiment). For each independent experiment, DCs (2 × 10^5^ cells/mL), macrophages (1 × 10^5^ cells/mL), and splenocytes (1 × 10^6^ cells/mL) were cultured in triplicate wells (technical triplicates) for 48 h with MPL (0.05 µg/mL), α-GalCer (0.05 µg/mL), or the combination (MPL+ α-GalCer); a vehicle control (Control) was included. Vehicle control – cells cultured in complete medium containing the same final DMSO concentration (0.005% v/v) as the combination-treated wells, without MPL or α-GalCer. Following incubation, cell-surface activation markers were analyzed by flow cytometry, and culture supernatants were collected for ELISA. All data represent N = 3 independent biological experiments performed on separate days, where each data point represents the mean of technical triplicates from a distinct cell preparation.

### Allogeneic T-cell proliferation co-culture

Primary DCs and macrophages were generated from BALB/c donor mice (n = 3 mice pooled per batch). Naïve allogeneic lymphocytes were isolated from C57BL/6 donor spleens (n = 3 mice pooled per batch). DCs and macrophages were pre-treated for 48 h with MPL (0.05 µg/mL), α-GalCer (0.05 µg/mL), or the combination (MPL+ α-GalCer). Naïve allogeneic lymphocytes were isolated from C57BL/6 spleens and labeled with carboxyfluorescein succinimidyl ester (CFSE). The pre-treated DCs (2 × 10^5^ cells/mL) or pre-treated macrophages (1 × 10^5^ cells/mL) were thoroughly washed three times to remove residual compounds and then co-cultured with CFSE-labeled lymphocytes (responder T cells) (2 × 10^6^ cells/mL) in 96-well U-bottom plates. Of note, responder T cell-only wells treated with adjuvant were not included as a control, so a residual contribution of carried-over compound to T cell proliferation cannot be formally excluded. After 5 days, cells were processed for flow cytometry to assess T-cell proliferation, and supernatants were retained for ELISA cytokine measurements. Data represent N = 3 independent biological experiments conducted on separate days using independent primary cell harvests.

### Statistical analysis

Data were analyzed using GraphPad Prism v9.2.0 and are expressed as mean ± SD. One-way ANOVA with Tukey’s post-hoc test was performed for Figs 1, 2, 3, 4C, 5, and 6. Two-way ANOVA with multiple comparisons was used for Fig 4A, and Kaplan-Meier analysis with the log-rank test was applied for Fig 4B. Statistical significance was set at *p < 0.05*. Exact *p*-values and test statistics are provided in S3 Table.

**Fig 3:**
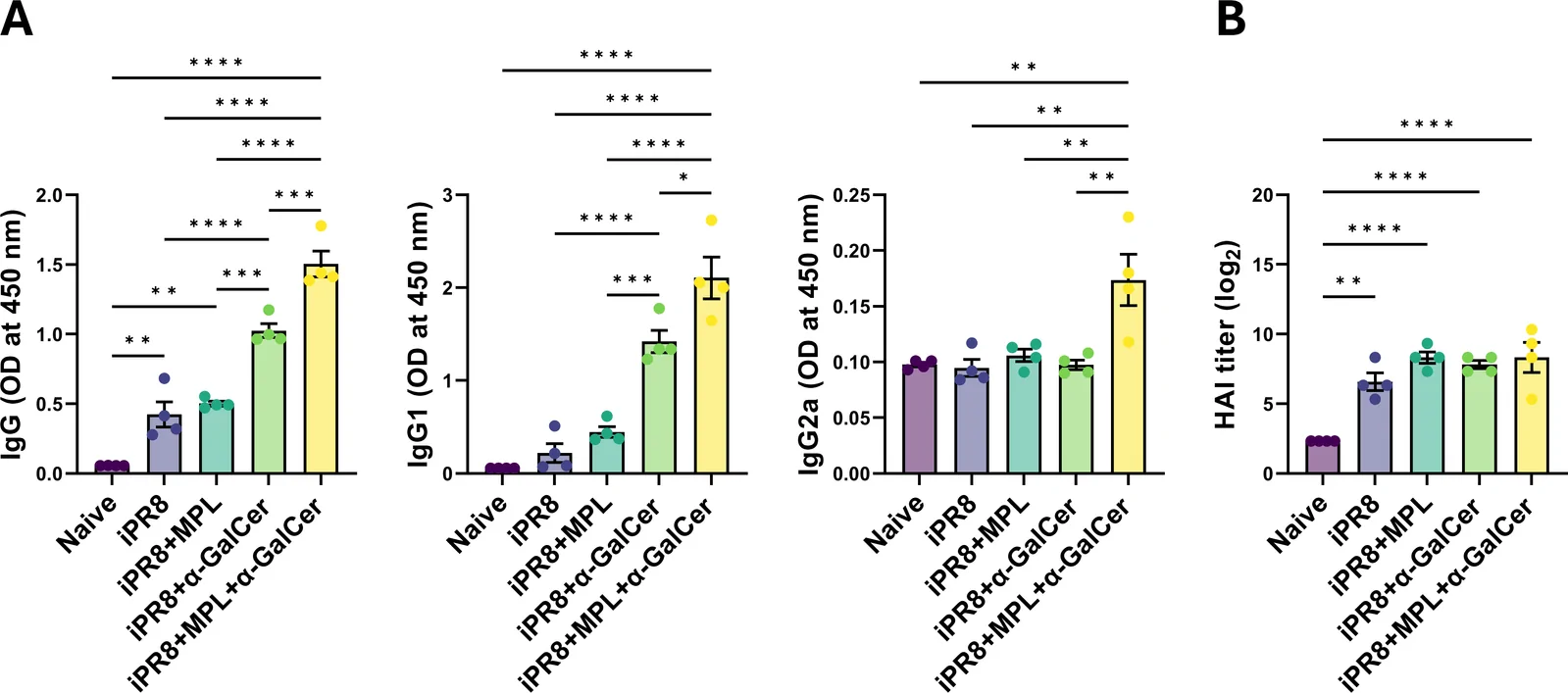
Antibody production following immunization with inactivated A/PR8 vaccine and adjuvants. BALB/c mice were given a prime intranasal immunization with inactivated A/PR8 (1 µg) either alone or adjuvanted with MPL (1 µg), α-GalCer (1 µg), or a combination (1 µg MPL + 1 µg α-GalCer). Immune sera were collected 2 weeks post-immunization from Cohort 2 (n = 4 mice/group), and A/PR8 virus-specific IgG antibody levels were measured by ELISA (A). Hemagglutination inhibition (HAI) titers in RDE-treated sera were determined two weeks after immunization (B). All data are expressed as the mean ± SD. Statistical analysis was performed using a one-way ANOVA followed by Tukey’s multiple comparisons test (*p < 0.05, **p < 0.01, ***p < 0.001, ****p < 0.0001 compare to indicated group).

**Fig 4:**
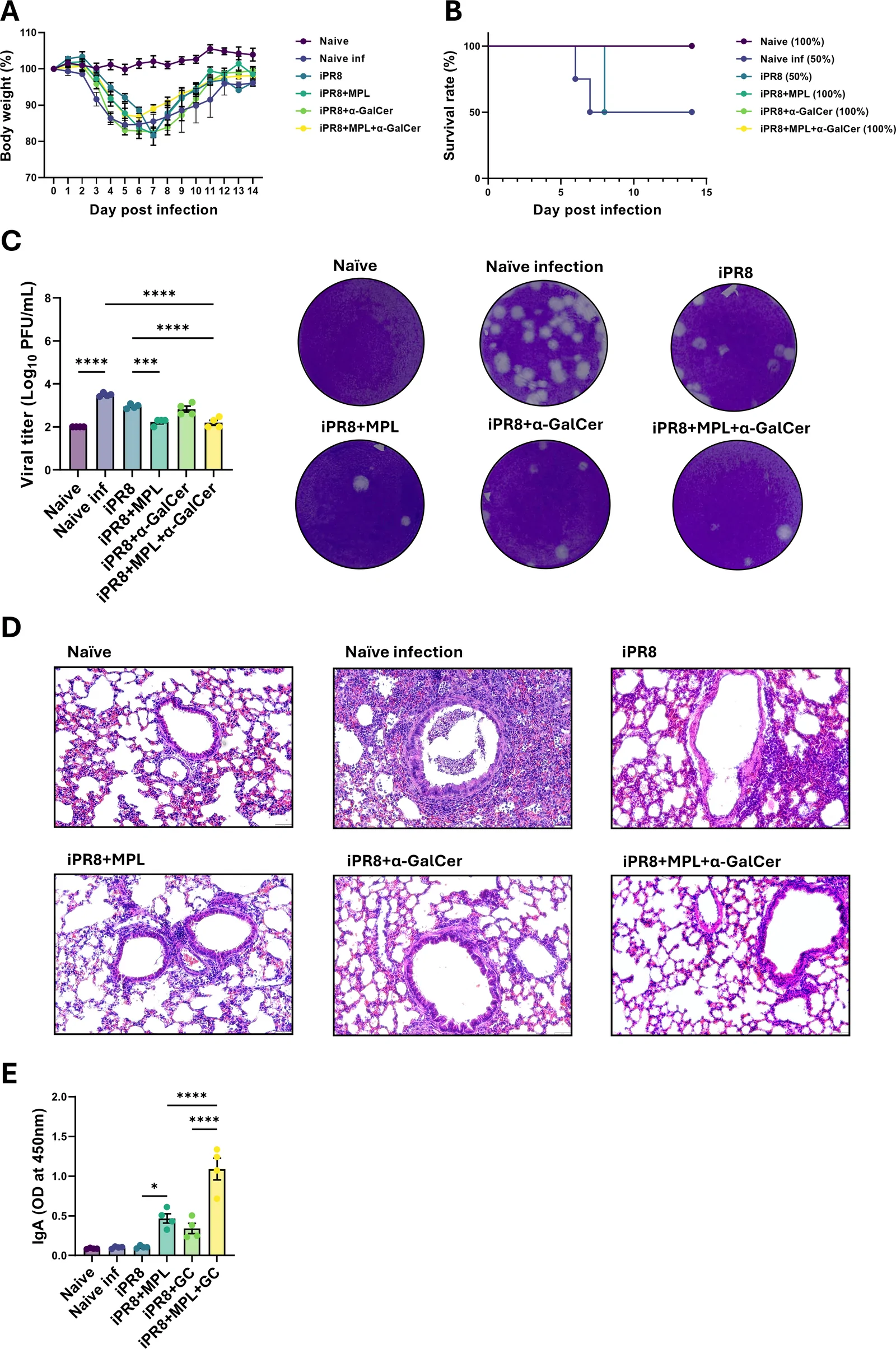
Protective efficacy and mucosal immune responses following A/PR8 challenge. Two weeks after immunization, naïve and immunized mice were infected with 100 PFU of the A/PR8 virus. (A) Body weight changes and (B) survival rates were monitored daily for 14 days post-infection in Cohort 1 (n = 4 mice/group). (C-E) In Cohort 2 (n = 4 mice/group), animals were sacrificed at 7 days post-infection (dpi) to determine lung viral titers by plaque assay (C), pulmonary histology by H&E staining (scale bar = 50μm) (D), and lung homogenate IgA levels by ELISA (E). All data are expressed as the mean ± SD. Statistical analysis was performed using Two-way ANOVA followed by Tukey’s multiple comparisons test (A), Kaplan-Meier analysis with the log-rank test (B), One-way ANOVA followed by Tukey’s multiple comparisons test (C, E) (*p < 0.05, **p < 0.01, ***p < 0.001, ****p < 0.0001 relative to the indicated group).

**Fig 5:**
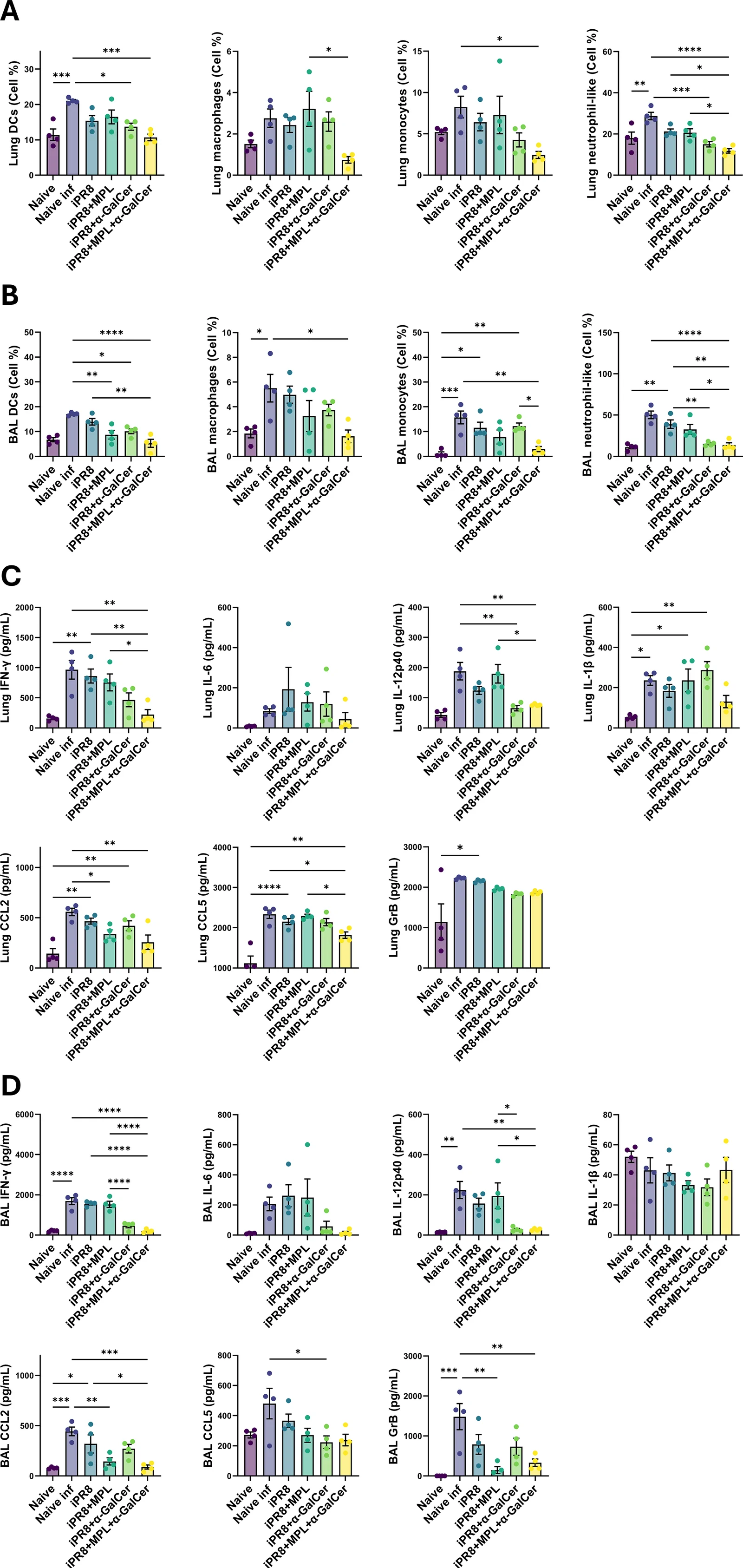
Cellular infiltration and cytokine/chemokine analysis in the lung and BAL fluid 7 days post-A/PR8 virus challenge. Two weeks after immunization, mice (n = 4 /group) were challenged with 100 PFU of the A/PR8 virus. Lung tissue and bronchoalveolar lavage (BAL) samples were harvested at 7 days post-infection from Cohort 2 (n = 4 mice/group). Cellular phenotypes within these samples were determined by flow cytometry (A, B). Cytokine and chemokine levels in lung (B) and BAL (C) fluid were quantified by ELISA. All data are expressed as the mean ± SD. Statistical analysis was performed using a one-way ANOVA followed by Tukey’s multiple comparisons test (*p < 0.05, **p < 0.01, ***p < 0.001, ****p < 0.0001 relative to the indicated group).

**Fig 6:**
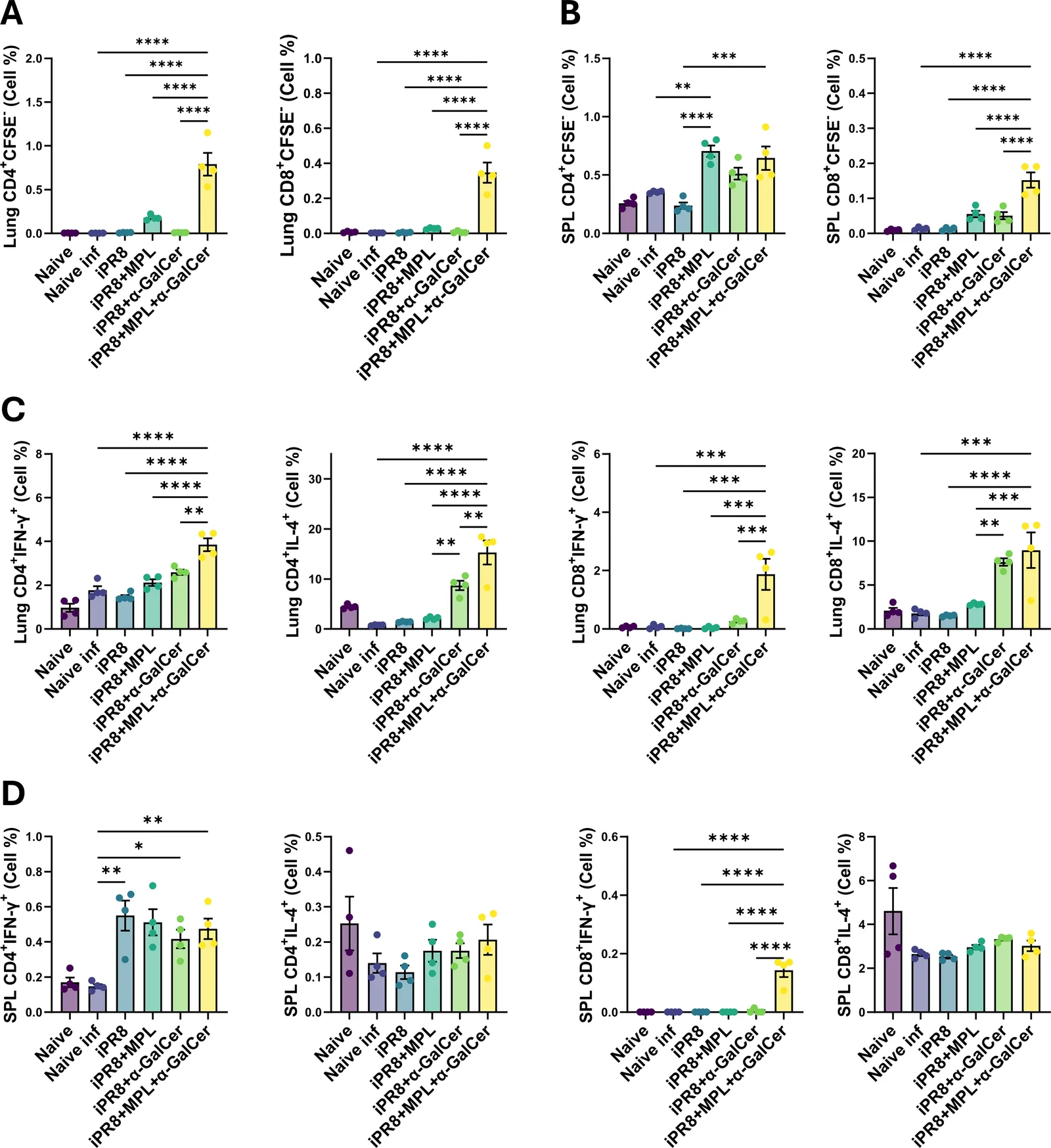
T-cell proliferation and IFN-γ and IL-4 producing T cells in the lung and spleen following *in vitro* iPR8 antigen stimulation. Lung and spleen cells were harvested at 7 days post-infection from Cohort 2 (n = 4 mice/group). Cells were labeled with CFSE and stimulated with inactivated PR8 (iPR8; 2 μg/mL) for 5 days, and T-cell proliferation was assessed by flow cytometry (A, B). For intracellular cytokine staining (ICS), lung and spleen cells were stimulated for 4 h (iPR8; 2 μg/mL) in the presence of GolgiStop, and cytokine-producing cells were analyzed by flow cytometry (C, D). All data are expressed as the mean ± SD. Statistical analysis was performed using a one-way ANOVA followed by Tukey’s multiple comparisons test (*p < 0.05, **p < 0.01, ***p < 0.001, ****p < 0.0001 relative to the indicated group).

## Results

1 **Combined MPL and α-GalCer adjuvant enhanced APC maturation and primes T-cell responses *in vitro***

To assess the *in vitro* immunostimulatory capacity of the adjuvants MPL (0.05 µg/ mL), α-GalCer (0.05 µg/ mL), and their combination (MPL+ α-GalCer), bone marrow-derived dendritic cells (DCs), macrophages, and splenocytes were cultured with the adjuvants for 48 hours. The activation status of the cells and cytokine secretion were then analyzed.

Treatment of MPL+ α-GalCer significantly increased the frequency of CD40^+^, CD86^+^, and MHC-II^+^ DCs and macrophages compared with vehicle control, reaching levels comparable to MPL alone, whereas α-GalCer alone showed no effect (Fig 1A, 1B). MPL+ α-GalCer also enhanced secretion of pro-inflammatory cytokines from APCs (TNF-α, IL-6, IL-12p40), again mirroring MPL and exceeding vehicle control.

In splenocyte cultures, the combination preferentially promoted T-cell activation (Fig 1C). Percentages of CD4^+^CD25^+^, CD8^+^CD25^+^, and CD4^+^CD69^+^ T cells significantly increased with MPL + α-GalCer treatment. CD49b^+^ T-cell subsets, described here as CD4^+^CD49b^+^CD25^+^ and CD4^+^CD49b^+^CD69^+^ NKT-like T cells, were also significantly expanded in the MPL+ GC group. By contrast, CD8^+^CD69^+^, CD8^+^CD49b^+^CD25^+^, and CD8^+^CD49b^+^CD69^+^ populations were unchanged. Splenocyte supernatants from the MPL+ α-GalCer group contained higher concentrations of TNF-α, IL-2, IFN-γ, IL-4 and IL-13 than vehicle-control cultures (Fig 1C).

Collectively, MPL+ α-GalCer is a potent *in vitro* activator of innate APCs maturation that concurrently primes adaptive T-cell responses.

2 **Combined MPL and α-GalCer primed APCs drive robust allogeneic T-cell proliferation and cytokine production**

To evaluate if adjuvant pre-activation of antigen-presenting cells (APCs) enhances their T-cell stimulatory capacity, DCs and macrophages were pre-treated with MPL (0.05 µg/ mL), α-GalCer (0.05 µg/mL), or their combination (MPL+ α-GalCer) and then co-cultured with allogeneic, CFSE-labeled lymphocytes.Flow-cytometric analysis showed that MPL+ α-GalCer pretreated DCs drove the highest proliferation of both CD4⁺ and CD8⁺ T cells, exceeding vehicle control and α-GalCer and matching or surpassing MPL alone (Fig 2A). Within both CD4⁺ and CD8⁺ subsets, MPL+ α-GalCer yielded the largest fractions of IFN-γ⁺ and IL-4 ⁺ cells (Fig 2B). Supernatants from DC co-cultures confirmed this profile, with IFN-γ, IL-4, and IL-13 highest in the MPL + α-GalCer treatment.

Macrophage stimulators showed a similar pattern. MPL+ α-GalCer-pretreated macrophages induced robust proliferation of CD4⁺ and CD8⁺ T cells and generated the greatest frequencies of IFN-γ⁺ and IL-4⁺ cells in both lineages (Fig 2C). Correspondingly, macrophage co-culture supernatants contained the highest levels of IFN-γ, IL-4, and IL-2 in the MPL+ α-GalCer group; granzyme B was significantly elevated with MPL+ α-GalCer and MPL versus vehicle control (Fig 2D).

Together, these data demonstrate that MPL+ α-GalCer pre-activation significantly enhances the ability of DCs and macrophages to drive robust T-cell proliferation.

3 **Single-dose intranasal MPL+ α-GalCer elicited the strongest PR8-specific IgG response**

To evaluate the humoral immune response, PR8-specific IgG antibody levels were measured in sera collected two weeks after a single intranasal immunization. Sera from mice receiving iPR8 (1 µg) +MPL (1 µg) +α-GalCer (1 µg) elicited the strongest antigen-specific IgG, IgG1 response compared with other treatment groups, with significantly enhanced antibody levels significantly higher A/PR8-specific IgG at a 1:100 serum dilution compared with that in the iPR8 alone group (Fig 3A). The immune response was also enhanced in the iPR8 + GC (1 µg) group compared to the iPR8 group, although not as effectively as in the MPL+ α-GalCer group. The iPR8 + MPL (1 µg) group produced only a modest rise, similar to the response in the iPR8 only group. Interestingly, only MPL+ α-GalCer adjuvant group increased the IgG2a level. Moreover, all vaccinated groups exhibited significantly higher HAI titers compared to the naïve group (Fig 3B). Specifically, the iPR8 + MPL + α-GalCer group and other adjuvanted groups achieved higher HAI activity compared to the group receiving iPR8 alone.

4 **MPL+ α-GalCer-adjuvanted iPR8 vaccine enhanced protection against influenza infection**

To evaluate the protective efficacy of the MPL+ α-GalCer adjuvanted vaccine, mice were challenged with 100 PFU of A/PR8 virus, a dose in the LD50 range. Infected naïve and iPR8-only mice exhibited severe weight loss and only 50% survival, whereas the iPR8+ MPL+ α-GalCer group showed the smallest weight drop, the fastest recovery, and 100% survival; the survival difference was not statistically significant (Fig 4A, 4B).

Histological evaluation of lung tissues from vaccinated mice demonstrated reduced cellular infiltration and alveolar septal thickening in these representative sections compared to the dense peribronchial inflammatory infiltrates observed in the naive-infection group (Fig 4D). This protection was mirrored by a dramatic reduction in lung viral titers (Fig 4C), with the iPR8+ MPL+ α-GalCer group reduced lung viral burden at day 7 post-infection. Furthermore, analysis at 7 days post-infection revealed that the MPL+ α-GalCer group maintained significantly elevated levels of post-challenge lung-associated antigen-specific IgA compared to other groups (Fig 4E).

Together, these results demonstrate that the MPL+ α-GalCer adjuvant was associated with enhanced systemic and mucosal responses, reducing lung viral burden alongside lower pulmonary inflammation.

5 **MPL +  α-GalCer-adjuvanted iPR8 vaccine: reduced lung viral burden accompanied by lower inflammatory cell infiltrates and cytokine/chemokine levels after A/PR8 challenge**

To relate protection to local inflammation, we quantified cells infiltrates (Fig 5A, 5B) and cytokine/chemokine levels (Fig 5C, 5D) in lung tissue and bronchoalveolar lavage (BAL) 7 days after A/PR8 challenge. Naïve-infected mice displayed the highest accumulation of inflammatory cells such as DCs, macrophages, monocytes, and neutrophil-like cells in both lung and BAL, accompanied by elevated pro-inflammatory cytokines (IFN-γ, IL-6, IL-12p40, IL-1β), chemokines (CCL2, CCL5), and granzyme B. Mice immunized with single-adjuvanted vaccines showed more inflammation than the MPL+ α-GalCer group. In contrast, MPL+ α-GalCer-adjuvanted iPR8 exhibited reduced cellular infiltration and significantly lower cytokine/chemokine levels in lung and BAL. These data indicate that MPL+ α-GalCer-adjuvanted iPR8 was associated with lower pulmonary inflammatory indices at this time point. Because these measurements were made at a single time point (7 dpi), at which lung viral titers differed markedly between groups, the lower inflammatory indices co-occurred with a reduced viral and antigen burden; the present design does not separate a burden-driven reduction in inflammation from a direct anti-inflammatory or immunoregulatory effect of the adjuvant combination.

6 **MPL +  α-GalCer-adjuvanted iPR8 vaccine enhanced post-challenge ex vivo antigen-responsive T-cell proliferation**

To evaluate antigen-specific T-cell recall responses following viral challenge, T-cell proliferation and cytokine production were analyzed in the lungs and spleen 7 days post-challenge. Mice immunized with the iPR8+ MPL+ α-GalCer displayed the highest frequencies of proliferating (CFSE⁻) CD4⁺ and CD8⁺ T cells in both the lungs and spleen after ex vivo iPR8 restimulation compared to the naïve and iPR8-only groups (Fig 6A, 6B). Furthermore, intracellular cytokine staining revealed that the MPL+ α-GalCer adjuvant significantly enhanced the functional capacity of these T cells, producing the highest level of IFN-γ⁺ and IL-4⁺ cells within both CD4⁺ and CD8⁺ T cells in the lungs (Fig 6C). A similar pattern was observed in the spleen, where the iPR8+ MPL+ α-GalCer group maintained elevated levels of cytokine-producing T cells, particularly IFN-γ⁺ CD8⁺ T cells, which were significantly higher than in all other groups (Fig 6D). These results demonstrate that the MPL+ α-GalCer combination increased the frequencies of ex vivo antigen-responsive, proliferating T cells”.

## Discussion

This study aimed to evaluate a novel intranasal vaccine adjuvant strategy by combining MPL, a TLR4 agonist, and α-GalCer, an NKT cell ligand, with an inactivated A/PR8 (iPR8) vaccine, showing that the MPL+ α-GalCer combination enhanced innate and adaptive immune responses, resulting in protection against homologous challenge that was accompanied by lower post-challenge pulmonary inflammatory indices.

Our *in vitro* investigation revealed the mechanistic basis for this enhanced combined response. In line with previous findings, MPL was the predominant driver of direct APCs maturation, significantly increasing the expression of co-stimulatory molecules (CD40, CD86) and MHC-II on both DCs and macrophages, whereas α-GalCer alone had no direct effect on these endpoints (Fig 1A, 1B). This confirms that initial APCs activation in the dual-adjuvant group is primarily MPL-driven [17]. However, the functional consequence of this activation was greatest with the combination. APCs pre-treated with MPL+ α-GalCer drove the most robust proliferation of allogeneic CD4⁺ and CD8⁺ T cells. While α-GalCer alone did not induce direct APC maturation *in vitro*, splenocytes exposed to MPL+ α-GalCer exhibited superior T-cell activation and cytokine production. Based on established literature, this complementary response may involve functional cross-talk between TLR4-activated APCs and glycolipid-responsive lymphocytes (such as NKT-like cells), wherein APC-derived IL-12 and lymphocyte-derived IFN-γ mutually reinforce T-cell priming (Fig 1C). This suggests that while MPL provides the primary signal for APCs maturation [19,27], α-GalCer-mediated activation (likely of NKT cells within the splenocyte cultures (Fig 1C)) contributes to an optimal cytokine environment that enhances T-cell priming [20,28].

The two adjuvants act on different cells through different receptors. MPL is a TLR4/MD-2 agonist signalling within the APC itself, so its programme is cell-intrinsic and fully expressed in purified BMDC and BMDM cultures [29]. α-GalCer is reported to have no receptor on DCs or macrophages: it is a CD1d-presented glycolipid whose target is the invariant TCR of iNKT cells, so its activity is expected to be cell-extrinsic and to depends on iNKT cells being present [30]. This would explains why α-GalCer alone produced no APC maturation in our cultures, which contain essentially no iNKT cells, yet matched MPL in splenocytes, where CD49b^+^ NKT-like cells are present. On this basis the two would be complementary rather than redundant, with MPL supplying signals 1 and 2, and α-GalCer supplying signal 3 and can license further DC maturation through CD40L-CD40.

Notably, the ranking of the two adjuvants differed between settings: MPL dominated every APC-based *in vitro* readout, whereas α-GalCer gave higher PR8-specific IgG and IgG1 and stronger T-cell responses *in vivo* at the same 1 µg dose. Four non-exclusive factors may account for this. First, the *in vitro* system contains almost none of the cells α-GalCer acts on iNKT cells are ~ 1–3% of splenic lymphocytes but 5–10% in lung and 10–30% in liver [31,32], and bone marrow-derived APC cultures contain essentially none, so a purified-APC assay has a structural blind spot for CD1d-dependent adjuvants, whereas intranasal delivery targets nasal-associated lymphoid tissue and lung, where iNKT cells are abundant. Second, α-GalCer may engage a B-cell help pathway without APC culture, in published studies show activated iNKT cells acquire a follicular helper phenotype and provide cognate help to CD1d-expressing B cells via CD40L, ICOS, IL-21 and IL-4, driving germinal-center formation and class switching, particularly to IgG1 [33–36], consistent with the pattern we observed, in which α-GalCer's advantage was greatest for IgG and IgG1. Third, the doses are not potency-equivalent: 0.1–2 µg per mouse is near-saturating for α-GalCer, whereas MPL is typically given at 10–50 µg per mouse in murine mucosal studies [37,38], while 0.05 µg/mL *in vitro* is saturating for TLR4. Fourth, MPL is rapidly cleared after mucosal delivery, whereas α-GalCer partitions into membranes and is presented on CD1d for a prolonged period. *In vitro* APC activation assays are therefore a valid but incomplete surrogate for mucosal adjuvant activity and should not be the sole screening criterion for ligands whose targets are tissue-resident innate-like lymphocytes.

The mixed cytokine profile observed *in vitro* was accompanied by a mixed IgG1/IgG2a antibody response *in vivo*. It is important to note that the combination group received a total adjuvant mass of 2 µg (1 µg MPL+ 1 µg α-GalCer) compared to 1 µg in the single-adjuvant groups. Therefore, the heightened downstream responses observed *in vivo* such as elevated serum IgG/IgG2a, lung IgA, and antigen-specific T-cell proliferative responses reflect an enhanced combined effect resulting from both the increased total adjuvant payload and the complementary activation of distinct cellular pathways (TLR4 on APCs and on the basis of the literature, CD1d on iNKT cells), rather than formal mathematical synergy.

Following a single intranasal immunization, the iPR8+ MPL+ α-GalCer group generated the strongest PR8-specific systemic IgG response, significantly outperforming all other groups (Fig 3). Critically, this was the only group to show a significant increase in the Th1-associated IgG2a subtype, alongside a strong Th2-associated IgG1 response. The induction of a balanced Th1/Th2 profile is particularly advantageous for influenza vaccines; Th1-driven responses are essential for efficient viral clearance, whereas Th2-driven responses ensure the production of high-titer neutralizing antibodies [39]. This balanced IgG1/IgG2a profile represents a more comprehensive form of humoral immunity compared to those induced by the vaccine alone or single-adjuvanted formulations. This antibody response was observed in the same groups that subsequently showed a reduced lung viral burden; no formal correlate-of-protection analysis was performed (Fig 4). A further observation is that protection in the iPR8+ MPL+ α-GalCer group was accompanied by lower pulmonary inflammatory indices following viral challenge. Influenza-associated morbidity and mortality are often driven by an excessive inflammatory response rather than the viral burden itself [40,41]. Reduced lung viral burden at 7 dpi in the iPR8+ MPL+ α-GalCer group was accompanied by lower inflammatory cell frequencies and lower cytokine and chemokine concentrations in lung and BAL fluid (Fig 5). Because both outcomes were measured in the same animals at the same time point, they cannot be separated mechanistically: the lower inflammation is at least as consistent with a reduced antigen and viral load driving less innate-cell recruitment and cytokine production as with a direct anti-inflammatory action of MPL+ α-GalCer. Moreover, distinguishing these possibilities would require viral-load-matched comparisons, sampling before viral titers diverge between groups, or depletion and CD1d-deficient models. This observation is consistent with the increased frequencies of ex vivo antigen-responsive T cells. Mice immunized with iPR8+ MPL+ α-GalCer displayed the highest frequencies of proliferating CD4⁺ and CD8⁺ T cells in both the lungs and spleen following antigen re-stimulation (Fig 6). Our findings are consistent with previous reports demonstrating that the co-administration of an α-GalCer analog and a TLR4 agonist significantly enhances CD8 ⁺ T-cell responses and provided enhanced protection against malaria [42]. Furthermore, the functional capacity of these cells was significantly enhanced, producing the highest levels of IFN-γ and IL-4 within the lung T-cell and IFN-γ⁺ CD8⁺ T cells in spleen.

A limitation of the present study is that canonical iNKT cells and CD1d dependence were not directly assessed: CD1d-α-GalCer tetramers were not included in the flow cytometry panels. Consequently, the CD3^+^CD49b^+^ (DX5^+^) populations analyzed in our experiments represent CD49b-expressing NKT-like T cells and activated conventional T cells rather than definitively isolated CD1d-restricted invariant NKT (iNKT) cells. While α-GalCer is a well-established iNKT cell agonist, further studies utilizing CD1d tetramer staining or CD1d-deficient animal models will be necessary to delineate the precise requirement of canonical iNKT cells in this dual-adjuvant system. This study was not designed as a safety evaluation; the absence of post-vaccination weight loss and of *in vitro* cytotoxicity indicates gross tolerability only, and a dedicated reactogenicity study including adjuvant-only groups and airway histopathology is required. Additionally, intranasal administration was performed using a 50 µL volume under anesthesia, a standard protocol in murine respiratory models. In anesthetized mice, this volume distributes across both the upper nasal passages and lower respiratory tract via pulmonary aspiration. Therefore, the protective immunity and reduced pathology reported here reflect combined upper and lower airway mucosal exposure rather than isolated nasal mucosal vaccination. This distinction is important for translational interpretation, as clinical human nasal sprays deliver formulations predominantly to the upper nasal cavity without significant lower airway deposition. Moreover, because viral challenge was conducted two weeks post-immunization, these findings highlight early protective efficacy during the effector phase. Determining the durability and spectrum of this protection will require future studies to evaluate immune contraction, memory cell longevity, heterologous cross-neutralization against drifted strains, upper-airway viral dynamics, and transmission.

Finally, the design constrains generality: all *in vivo* work used female BALB/c mice (C57BL/6 served only as *in vitro* allogeneic responders), and the two strains differ in Th1/Th2 bias and iNKT subset composition; each adjuvant was tested at a single dose with no dose-response; a single dose, route and challenge time point were examined; both vaccine antigen and challenge virus were A/PR/8/34, so heterologous protection, breadth, upper-airway replication and transmission were not assessed; and n = 4 per group in vivo with N = 3 *in vitro* gives limited statistical power. These findings should be regarded as a proof of concept requiring confirmation across additional strains, doses, regimens and viruses. Inflammatory endpoints were also assessed only at 7 dpi, when lung viral titers already differed substantially between groups; the lower inflammatory-cell frequencies and cytokine/chemokine concentrations in the MPL+ α-GalCer group are therefore confounded by viral burden, and a direct immunoregulatory effect of the adjuvant combination was not demonstrated.

## Conclusion

In a short-term homologous PR8 mouse challenge model, intranasal administration of inactivated PR8 with MPL and α-GalCer was associated with enhanced systemic antibody responses, lower lung viral titers at 7 days post-infection, and reduced post-challenge inflammatory indices compared with several comparator groups. The study does not establish statistical synergy, iNKT-cell dependence, durable immunological memory, or a causal role for pre-existing mucosal IgA. These findings support the MPL+ α-GalCer combination as a candidate mucosal adjuvant pair warranting evaluation in adequately powered studies incorporating dose-matched controls, CD1d-tetramer-based identification of iNKT cells, pre-challenge mucosal sampling, multi-time-point virological kinetics, and heterologous challenge.

## Supporting information

S1 Fig Verification of complete inactivation of the iPR8 vaccine antigen. Confluent Madin-Darby canine kidney (MDCK) cell monolayers were left untreated (Control), infected with live A/PR/8/34 (PR8) virus at a multiplicity of infection (MOI) of 0.01, or incubated with formalin-inactivated PR8 (iPR8; 1 µg/mL). Monolayers were examined by phase-contrast light microscopy 3 days after inoculation. Live PR8 produced an extensive cytopathic effect (CPE), evident as cell rounding, detachment and disruption of the monolayer, whereas monolayers exposed to iPR8 remained intact and indistinguishable from the untreated control, confirming that no residual infectious virus remained after formalin inactivation. No serial (blind) passage was performed. Scale bar = 100 µm.
(TIF)

S2 Fig Adjuvant treatment does not compromise *in vitro* cell viability or post-immunization body weight. (A) Bone marrow-derived dendritic cells (DCs), bone marrow-derived macrophages and splenocytes were cultured for 48 h with vehicle (Control), MPL (0.05 µg/mL), α-GalCer (0.05 µg/mL) or MPL + α-GalCer. Cell viability was determined colorimetrically and is expressed as absorbance at 450 nm (N = 3 independent cultures). (B) Female BALB/c mice (n = 4/group) received a single intranasal immunization with iPR8 (1 µg) alone or adjuvanted with MPL (1 µg), α-GalCer (1 µg) or MPL + α-GalCer (1 µg each). Body weight was recorded daily for 7 days after immunization.
(TIF)

S3 Fig Representative flow cytometry plots of *in vitro* DCs and macrophage activation. Bone marrow-derived DCs (A) and macrophages (B) were cultured for 48 h with vehicle (Control), MPL (0.05 µg/mL), α-GalCer (0.05 µg/mL) or MPL + α-GalCer, and surface expression of CD40^+^, CD86^+^ and MHC-II^+^ was assessed by flow cytometry. Each plot shows marker fluorescence intensity versus FSC-H, with the gate delineating marker-positive cells. Columns, treatment groups; rows, activation markers. Plots are representative for the summary data in Fig 1A and 1B. The gating strategy is shown in S7 Fig.
(TIF)

S4 Fig Representative flow cytometry plots of *in vitro* splenocyte activation. Splenocytes were cultured for 48 h with vehicle (Control), MPL (0.05 µg/mL), α-GalCer (0.05 µg/mL) or MPL + α-GalCer, and activation of conventional and CD49b^+^ (NKT-like) T-cell subsets was assessed by flow cytometry. Rows (top to bottom): CD4^+^CD25^+^, CD4^+^CD69^+^, CD8^+^CD25^+^, CD8^+^CD69^+^, CD4^+^CD49b^+^CD25^+^, CD4^+^CD49b^+^CD69^+^, CD8^+^CD49b^+^CD25^+^ and CD8^+^CD49b^+^CD69^+^; columns, treatment groups. Plots are representative and correspond to the summary data in Fig 1C. The gating strategy is shown in S8 Fig. Because CD1d-α-GalCer tetramers were not included in the panel, CD3^+^CD49b^+^ cells represent CD49b-expressing NKT-like and activated conventional T cells rather than definitively CD1d-restricted invariant NKT cells.
(TIF)

S5 Fig Representative flow cytometry plots for the mixed lymphocyte reaction with adjuvant-pre-treated dendritic cells. Bone marrow-derived DCs from BALB/c mice were pre-treated for 48 h with vehicle (Control), MPL, α-GalCer or MPL + α-GalCer (0.05 µg/mL each), washed thoroughly, and co-cultured for 5 days with CFSE-labelled allogeneic splenic lymphocytes from C57BL/6 mice. Rows (top to bottom): CD4^+^CFSE^-^, CD4^+^CFSE^-^IFN-γ^+^, CD4^+^CFSE^-^IL-4^+^, CD8^+^CFSE^-^, CD8^+^CFSE^-^IFN-γ^+^ and CD8^+^CFSE^-^IL-4^+^ cells; columns, pre-treatment groups. CFSE- denotes proliferating (CFSE-diluted) cells. Plots are representative and correspond to the summary data in Fig 2A and 2B. The gating strategy is shown in S9 Fig.
(TIF)

S6 Fig Representative flow cytometry plots for the mixed lymphocyte reaction with adjuvant-pre-treated macrophages. Bone marrow-derived macrophages from BALB/c mice were pre-treated for 48 h with vehicle (Control), MPL, α-GalCer or MPL + α-GalCer (0.05 µg/mL each), washed thoroughly, and co-cultured for 5 days with CFSE-labelled allogeneic splenic lymphocytes from C57BL/6 mice. Rows (top to bottom): CD4^+^CFSE^-^, CD4^+^CFSE^-^IFN-γ^+^, CD4^+^CFSE^-^IL-4^+^, CD8^+^CFSE^-^, CD8^+^CFSE^-^IFN-γ^+^ and CD8^+^CFSE^-^IL-4^+^ cells; columns, pre-treatment groups. Plots are representative of three independent experiments and correspond to the summary data in Fig 2C and 2D. The gating strategy is shown in S9 Fig.
(TIF)

S7 Fig Gating strategy for *in vitro* dendritic cell and macrophage activation. Cultured cells were sequentially gated on the total cell population (FSC-A versus SSC-A), single cells (FSC-A versus FSC-H) and live cells (LIVE/DEAD™ Fixable Aqua-negative). Macrophages were then identified as CD11b^+^ and dendritic cells as CD11c^+^. Within each population, expression of the activation markers CD40, CD86 and MHC-II was quantified (bottom row).
(TIF)

S8 Fig Gating strategy for in vitro T-cell and NKT-like cell activation. Splenocytes were sequentially gated on the total cell population (FSC-A versus SSC-A), single cells (FSC-A versus FSC-H), live cells (LIVE/DEAD™ Fixable Aqua-negative), CD45^+^ leukocytes and CD3^+^ T cells. CD49b (clone DX5) expression was used to separate conventional T cells (CD49b-negative) from CD49b^+^ NKT-like T cells, and each fraction was further resolved into CD4^+^ and CD8^+^ subsets. Activation was assessed by CD25^+^ and CD69^+^ expression (bottom row).
(TIF)

S9 Fig Gating strategy for T-cell proliferation and intracellular cytokine staining. CFSE^-^labelled lymphocytes were sequentially gated on the total cell population (FSC-A versus SSC-A), single cells (FSC-A versus FSC-H), live cells (LIVE/DEAD™ Fixable Aqua-negative), CD45^+^CD3^+^ T cells and then CD4^+^ and CD8^+^ subsets. Proliferating cells were identified as CFSE-diluted (CFSE^-^), and IFN-γ^+^ and IL-4^+^ cells were enumerated within each T-cell subset.
(TIF)

S10 Fig Gating strategy for inflammatory cell infiltrates in the lung and bronchoalveolar lavage fluid. Single-cell suspensions from lung tissue and bronchoalveolar lavage (BAL) fluid were sequentially gated on the total cell population (FSC-A versus SSC-A), single cells (FSC-A versus FSC-H), live cells (LIVE/DEAD™ Fixable Aqua-negative) and CD45^+^ leukocytes and were then separated into CD11b^+^ and CD11b^-^ fractions. Within the CD11b^+^, macrophages were identified as F4/80^+^, and the F4/80^-/low^ fraction was resolved by Ly6C into Ly6C^high^ monocytes and Ly6C^intermediate^ neutrophil-like cells, because Ly6G was not included, this population is termed “neutrophil-like” rather than neutrophils. Within the CD11b^-^, dendritic cells were identified as F4/80^-^CD11c^+^MHC-II^+^.
(TIF)

S1 Table Antibodies used for flow cytometry. (XLSX)

S2 Table Commercial ELISA kits used for cytokine and chemokine quantification. (XLSX)

S3 Table Exact p-values and statistical tests for all comparisons shown in Figs 1–6. Exact multiplicity-adjusted p-values reported by GraphPad Prism for every pairwise comparison presented in Figs 1–6, together with the parameter evaluated and the statistical test applied. Significance: *p < 0.05, **p < 0.01, ***p < 0.001, ****p < 0.0001; ns, not significant (p ≥ 0.05). Values below 0.0001 are reported as <0.0001 and values above 0.9999 as >0.9999. All data are expressed as the mean ± SD.
(XLSX)

S4 Table Underlying numerical data for Figs 1–6 and S2 Fig. (XLSX)
